# Outcome of multi-staged induced membrane technique based on post-debridement cultures for the management of critical-sized bone defect following fracture-related infection

**DOI:** 10.1038/s41598-022-26746-2

**Published:** 2022-12-31

**Authors:** Jae-Woo Cho, William T. Kent, Jin-Kak Kim, Seong-Ho Jeong, Seungyeob Sakong, Hanju Kim, Whee Sung Son, Eic Ju Lim, Wonseok Choi, Jong-Keon Oh

**Affiliations:** 1 Department of Orthopaedic Surgery, Korea University Guro Hospital, Korea University Medicine, 148, Gurodong-Ro, Guro-Gu, 08308 Seoul, Republic of Korea; 2 Department of Orthopaedic Surgery, University of California, San Diego, CA USA; 3 Department of Orthopaedic Surgery, Seoul National University Bundang Hospital, Kyungki, Republic of Korea; 4 Department of Plastic Surgery, Korea University Guro Hospital, Korea University Medicine, Seoul, Republic of Korea; 5 Department of Orthopaedic Surgery, Chungbuk National University Hospital, Chungbuk National University College of Medicine, Cheongju, Republic of Korea

**Keywords:** Trauma, Outcomes research

## Abstract

The authors’ institution utilizes multi-staged induced membrane technique protocol based on post-debridement culture in treating patients with critical-sized bone defect in lower extremity due to infected nonunion or post-traumatic osteomyelitis. This study aimed to evaluate the success rate of this limb reconstruction method and which risk factors are associated with recurrence of infection. 140 patients were treated with multi-staged induced membrane technique from 2013 to 2018 and followed up more than 24 months after bone grafting. The primary success rate of limb reconstruction was 75% with a mean follow-up of 45.3 months. The mean Lower Extremity Functional Scale in success group improved from 12.1 ± 8.5 to 56.6 ± 9.9 after the treatment. There were 35 cases of recurrence of infection at a mean of 18.5 months after bone grafting. Independent risk factors for recurrence of infection were infected free flap, surprise positive culture, deviation from our surgical protocol, and elevated ESR before final bone graft procedure. In conclusion, this study showed that multi-staged induced membrane technique protocol based on post-debridement culture resulted in 75% success rate and revealed a number of risk factors for recurrence of infection.

## Introduction

The induced membrane technique (IMT), which includes debridement and placement of polymethyl methacrylate (PMMA) cement during the first stage, followed by autogenous bone grafting once the osteoinductive membrane chamber has formed during the second stage, has been used to address critical-sized bone defects (CSBDs)^1,2^. Additionally, this technique has been applied and modified to treat chronic bone infections including the treatment of infected nonunions (IN) and post-traumatic osteomyelitis (PTOM)^3,4^.

Although several clinical studies on the biologically active membrane’s ability to consolidate grafted bone have yielded favorable results, high rates of recurrence of infection(ROI) have been reported following the staged reconstruction protocol^5,6^. With few large homogenous series using a standardized IMT protocol for the treatment of IN and PTOM, there is little evidence to support their effectiveness in treating these often challenging infections.

Our institution has treated recalcitrant IN and PTOM using a post-debridement culture (PDC)-based, multi-staged IMT protocol since 2013^7,8,9^. In light of the above, we aimed to answer the following research questions: (1) What is the success rate of limb reconstruction in patients with CSBD of the lower extremity due to IN or PTOM using our PDC-based, multi-staged IMT protocol and (2) What risk factors are associated with ROI in this population?

## Material and methods

### Design and settings

We retrospectively analyzed a cohort of patients diagnosed with Fracture- related Infection (FRI) criteria of the lower extremity from January 2013 to 2018 at a trauma fellowship training university hospital^10^. The limb reconstruction team in hospital have been operated by two orthopaedic trauma fellowship-trained surgeons, one microsurgery trained plastic surgeon and one hand and microsurgery trained orthopaedic surgeon.

The patients had already undergone unsuccessful surgical treatment for infection of their IN or PTOM at least twice at an outside hospital and presented to our institution with clinical, laboratory, and radiographic findings all consistent with recalcitrant Fracture-related Infections^11,12^. Patients who underwent a PDC-based, multi-staged IMT protocol for the treatment of IN or PTOM and post-debridement CSBD reconstruction of the femur or tibia (critical sized bone defect was defined as ‘segmental defects of at least 2.5 cm in longitudinal axis or more than 50% of circumferential wedge bone defect’) were enrolled. We excluded patients with affected upper extremities, those who were treated using other modalities, including primary amputation and distraction osteogenesis.

**Figure 1:**
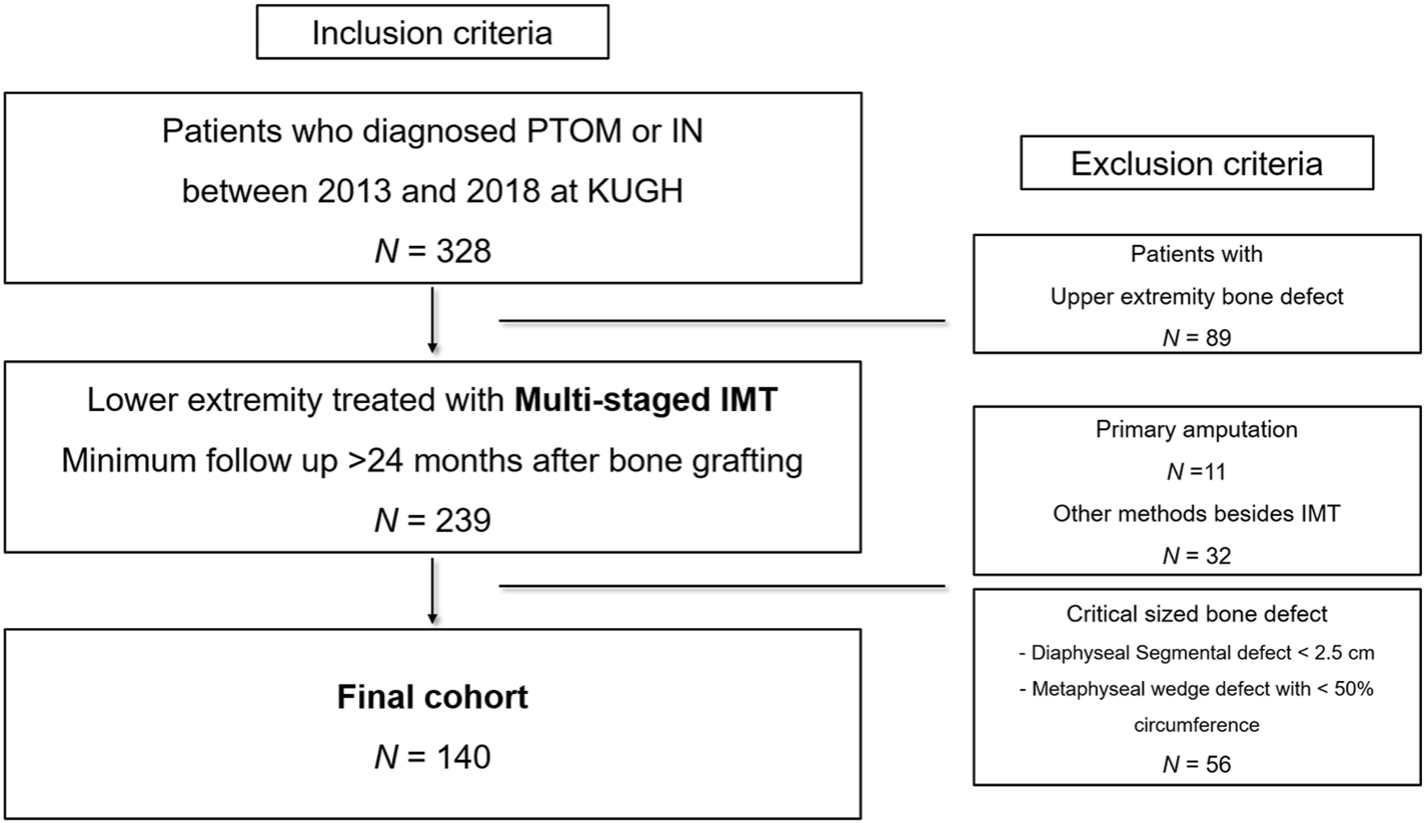
Final cohort based on inclusion and exclusion criteria.

Among 239 patients who were treated by recalcitrant IN or PTOM in the lower extremity and followed up at least more than 24 months, 11 patients who were treated by primary amputation, 24 patients who were treated by distraction osteogenesis, 8 patients who did not want to reach final bone grafting then, had the defect filled with permanent cement and 56 patients whose defect before bone grafting was not matched with the radiologic criteria of critical size bone defect were excluded. A total of 140 patients were consisted of the final cohort (Fig. 1).

### PDC based, multi-staged IMT protocol

**Figure 2:**
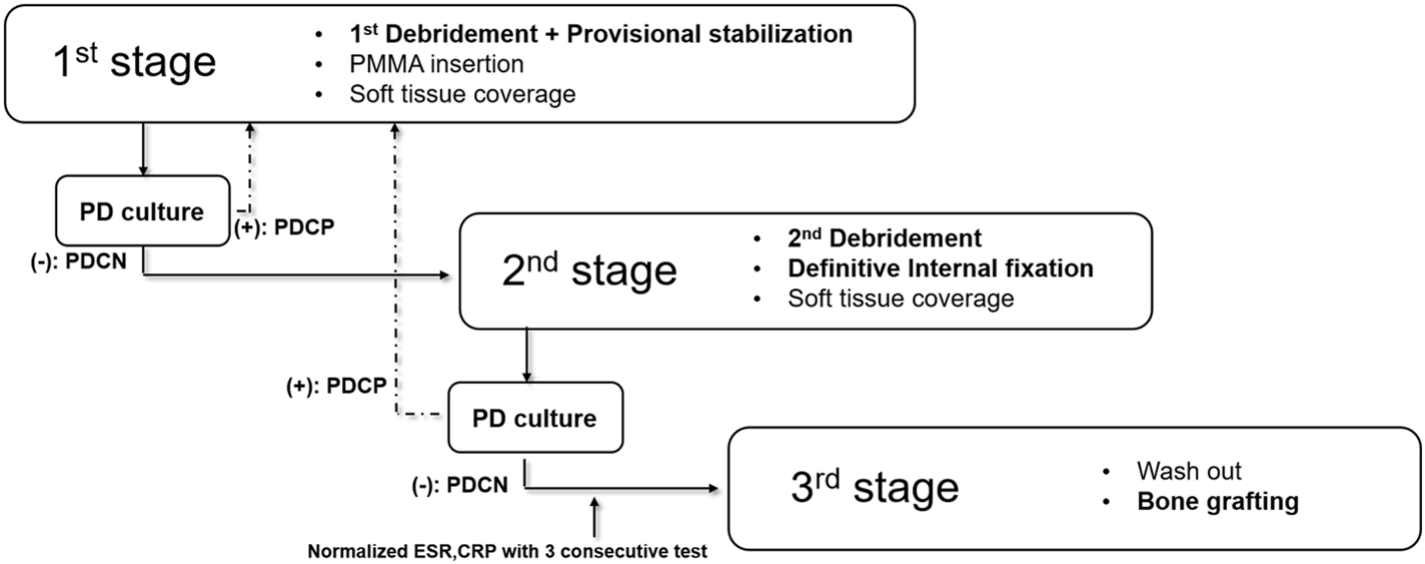
Author’s PDC based multi-staged induced membrane technique. The first stage involved thorough debridement and antibiotics loaded cement spacer insertion, followed by temporary stabilization using an external fixator, temporary plating, an antibiotic-loaded cement rod, or a combination thereof. The second stage included secondary debridement and conversion to the definitive fixation construct using an antibiotic-loaded PMMA cement spacer. The final stage involved removing the cement spacer and bone grafting once the infection was eradicated.

Our surgical protocol is based on the IMT, including multiple stages of reconstruction to address recalcitrant recurrent infections as per the aforementioned protocol^7,8,9^. The first stage involved thorough debridement and antibiotics loaded cement spacer insertion (vancomycin 4 g + , tobramycin 0.4 g per 40 g PMMA), followed by temporary stabilization using an external fixator, temporary plating, an antibiotic-loaded cement rod, or a combination thereof. The second stage included secondary debridement and conversion to the definitive fixation construct using an antibiotic-loaded PMMA cement spacer. In this stage, long bone realignment was achieved with nailing, plating, or a combination of methods. The final stage involved removing the cement spacer and bone grafting once the infection was eradicated. We confirmed the latter using serial serologic examination, including ESR and CRP. Clinical manifestations in the donor site were investigated via outpatient follow-up every 2–3 months. If soft tissue was infected or too fragile to cover the defect area, coverage with a fasciocutaneous or muscle flap was implemented at the first or second debridement stage before the final bone grafting (Fig. 2).

**Figure 3:**
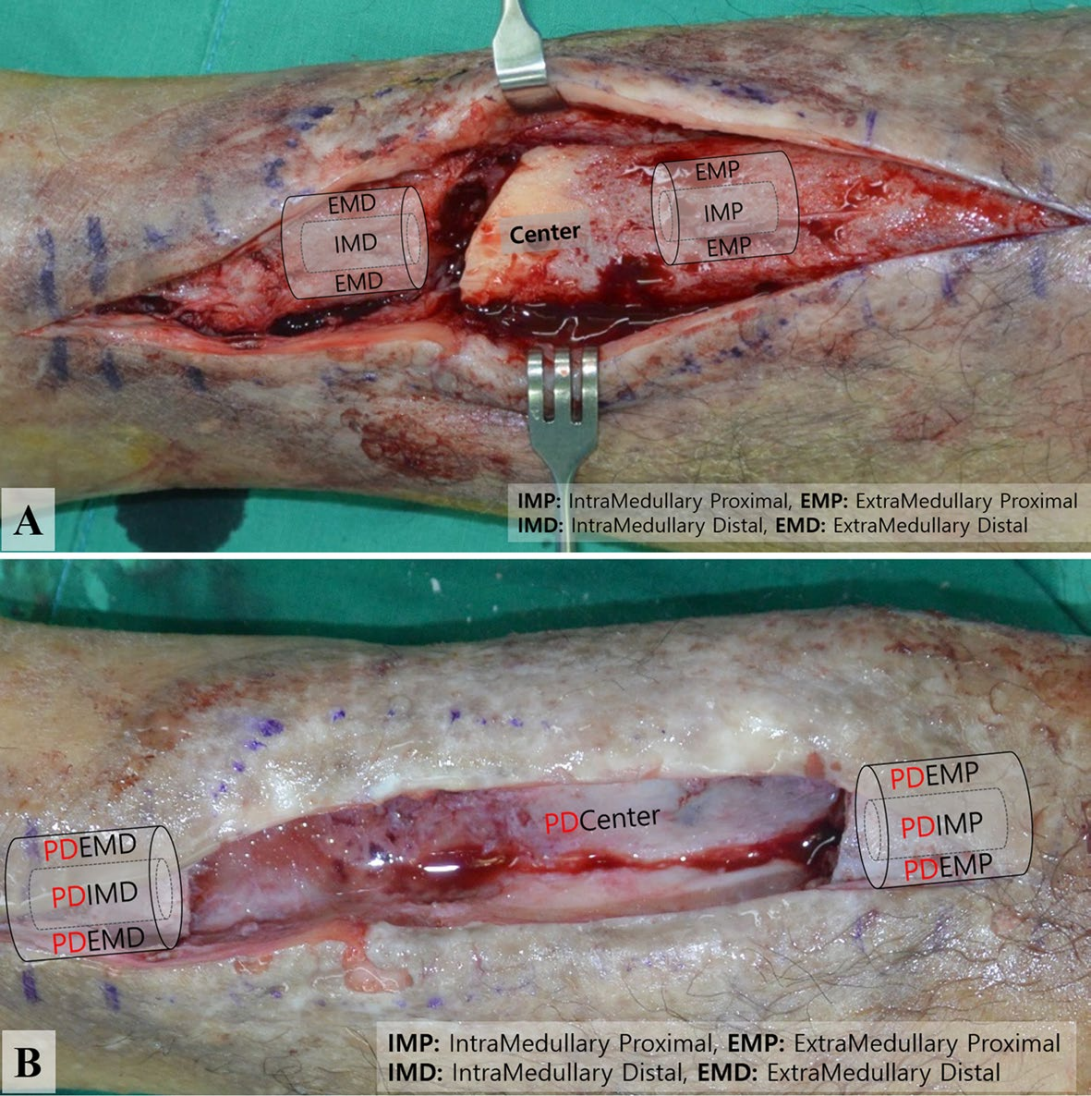
Intraoperative site-specific culture, including both index (**A**) and PD cultures (**B**).

During the staged surgical protocol, we systematically assessed site-specific tissue cultures collected intraoperatively to identify the associated microorganisms. At each stage of the protocol (stages 1–3), tissue cultures were obtained. At least five intraoperative tissue cultures were taken, beginning at the center of the infected bony lesion and out to the periphery during the debridement stage of the procedure (defined as: center, intramedullary proximal, intramedullary distal, extramedullary proximal, and extramedullary distal). The first culture set was named the index culture. Copious irrigation with normal saline followed thorough debridement. Next all drapes and contaminated instruments were removed from the field and changed and post debridement cultures (PDC) were obtained in a similar fashion as the index cultures (5 cultures were obtained starting at the center of the lesion working to the periphery) (Fig. 3). With our staged protocol, negative PD cultures (PDCN) were required to proceed to the next stage. When the PD culture was identified as positive (PDCP), we repeated the previous stage with irrigation and debridement with additional cultures (both index and post debridement) obtained from the site. Whenever there was hardware, sonication fluid culture was taken to increase the detection rate of microorganism.

### General antibiotic protocol

**Figure 4:**
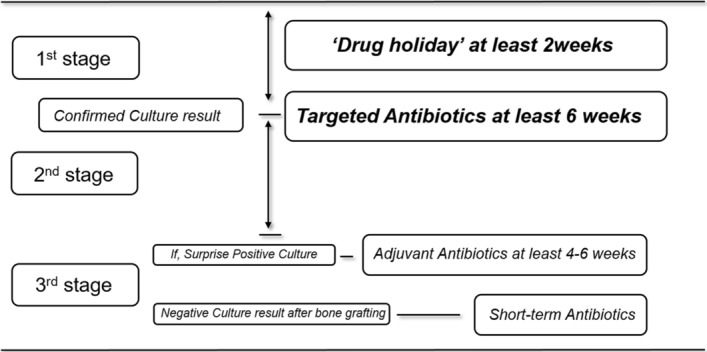
Antibiotics protocol during multi-staged IMT. All patients received at least 2 weeks of an antibiotic drug holiday prior to being engaged in the protocol. Once we identified the microorganisms from the initial debridement, targeted intravenous antibiotics were administered under the guidance of an infectious disease specialist. Intravenous antibiotics were administered for at least 6 weeks. “Surprise culture positive” cases were those in which a positive index culture result followed a negative post-debridement culture result from the previous stage. In such cases, adjuvant targeted IV antibiotics were administered for at least 4 or 6 weeks from the time of last positive culture.

All patients received at least 2 weeks of an antibiotic drug holiday prior to being engaged in the protocol. Once we identified the microorganisms from the initial debridement, targeted intravenous antibiotics were administered under the guidance of an infectious disease specialist. Intravenous antibiotics were administered for at least 6 weeks. “Surprise culture positive” cases were those in which a positive index culture result followed a negative post-debridement culture result from the previous stage^13^. In such cases, adjuvant targeted IV antibiotics were administered for at least 4 or 6 weeks from the time of last positive culture (Fig. 4).

### Study variables

Patient demographics, intraoperative findings, and clinical follow-up data were collected prospectively and analyzed retrospectively. Patient demographics, including age, sex, body mass index, and medical comorbidities, were documented.

The severity of infection, including duration and details regarding previous infection control surgeries, were analyzed by medical chart review and previous radiographs. The location of infection was subdivided using AO classification^14^. The type of bone infection was classified using the Cierny-Mader classification^15^.

During the staged operations, microorganisms grown from intraoperative cultures were documented. Patients were divided into culture-negative, monomicrobial, and polymicrobial infections. Anaerobic microorganisms, gram-negative microorganisms, and pseudomonal infections were documented as independent variables.

We analyzed the bone grafting environment, including the size (< 50 mm, 50-100 mm or > 100 mm) and type of bone defect (segmental type or wedged type); condition of the surrounding soft tissue and associated major vessels; number of debridement procedures before the final bone grafting; membrane duration; and fixation construct type. Details regarding the bone grafting, including the properties of the graft material (pure autogenous bone grafting or mixed grafting with demineralized bone matrix (DBM)), method of grafting, addition of bone morphogenetic protein 2, and operation time, were analyzed. Serologic markers, including Erythrocyte Sedimentation Rate (ESR) and C-Reactive Protein (CRP) prior to bone grafting, were documented.

Adherence to the surgical protocol was analyzed as a variable. Cases which diverted from the standardized protocol were termed “deviation of protocol.” These included instances where (1) protocol stages were combined or overlapped, (2) occasions where we did not revert back to a previous stage after positive cultures (e.g. PDCP from a previous stage) and (3) PDCP at second-stage treated with debridement and implant retention (where normal protocol calls for removal of previously placed implants in a PDCP). These three reasons for deviation of the protocol were all examined.

### Outcome measures

The primary endpoint of this study was the success rate of limb reconstruction after our staged IMT for the treatment of IN and PTOM. We defined a successful reconstruction as no ROI at minimum 2-year follow-up after the final bone grafting with bony consolidation of the CSDB. The bony consolidation was evaluated with consecutive CT scans and determined by one independent observer who has completed an orthopaedic trauma fellowship. In addition, the functional outcomes before and after the treatment were evaluated at first visit and last follow up using the lower extremity functional scale (LEFS)^16,17^.

ROI was diagnosed with Fracture- related Infection (FRI) criteria^10^. When ROI was suspected based on clinical manifestations, such as erythema, swelling, pain, and elevated laboratory values including serum white blood cell count, and inflammatory markers, the final diagnosis was confirmed by intraoperative culture results during subsequent infection control surgery. The secondary endpoint of this study was to identify the risk factors associated infection recurrence.

### Statistical analysis

Statistical analysis was performed using the SPSS software package (SPSS, Chicago, IL, USA). We performed a descriptive analysis of the epidemiological and morphological factors of the cohort. Survival analysis was performed using Kaplan–Meier curves, and ROI was defined as the end-point. To determine the risk factors associated with ROI, multivariate Cox regression analysis was performed using the variables with *p* values < 0.05, as determined using two-tailed tests.

### Ethical approval

All experimental protocols were approved by Korea University Guro Hospital Institutional Review Board. All procedures and methods performed in studies involving human participants were in accordance with the ethical standards of the institution or practice at which the studies were conducted. (IRB number: 2021GR0003) Informed consent was obtained from all individual participants included in the study.

## Results

One hundred fifteen male and 25 female patients with a mean age of 52.3 (range, 20–87) years, with a total of 43 femurs and 97 tibias were included in the study. The mean number of previous infection control surgeries at other hospitals prior to presenting to our institution was 3.5 (range, 2–18), while the mean infection duration before introduction of our protocol was 49.3 months (range, 2–239 months).

Eighty-one patients had monomicrobial infections, 39 patients had polymicrobial, and 20 patients had culture-negative infections. The mean length of the defects was 90.1 mm (range 30.3–219.0 mm). There were 110 segmental defects and 30 wedge type defects. Among the 70 flap-covered defects, 23 were performed at other hospitals and were found to have residual infection during the multi-staged IMT protocol. We used the term “infected previous free flap” for analysis.

Prior to bone grafting, the mean number of times patients underwent debridement and fixation surgery was 2.5 (range 1–7). The mean duration of the membrane at bone grafting was 129.5 days (range 42–375 days). The defects were stabilized using intramedullary nails in 69 patients, plating in 34 patients, and combined plate/nail fixation in 37 patients. Pure autogenous bone grafting alone was used in 70 patients, while mixed bone grafting with DBM (ALLOMIX®, CGBIO) was performed in other 70 patients. There was a deviation in protocol in 41 patients. This included combining stages of the protocol in 10 patients, 22 patients where the decision was made not to return to a previous stage in the protocol after a PDCP from a previous stage, and 9 patients who had retention of implants after PDCP. An elevated ESR at the time of bone grafting was observed in 84 patients (Tables 1, 2).

**Table 1:** Demographic, location and severity of infection, Microorganism, Environment of bone grafting, Details of bone grafting, and surgical procedure in 140 patients.

Variables	N or mean	Range	SD	SE	95% C.I
**Patient demographics**					
Age	52.3 yrs	20–87	15.1	1.32	49.94–55.15
Sex	Female: 25				
	Male: 115				
ASA classification	I: 47				
	II: 70				
	III: 19				
	IV: 4				
Previous smoking history	Smoker: 64				
	Non-Smoker: 76				
Diabetes	23 pts				
BMI	25.6 kg/m^2^	19.3–38.8	3.6	0.3	24.99–26.17
Normal < 25	68				
25 < Overweight < 30	58				
Obesity > 30	14				
**Location and severity of infection**					
Location of infection	Femur: 43 cases				
	32:23				
	33:14				
	33 with knee joint involvement: 6				
	Tibia : 97 cases				
	41:21				
	42:43				
	43:19				
	43 with ankle joint involvement : 14				
Duration of infection	49.3 months	3–239	27.2	14.4	44.8–53.8
< 3 month:	24				
3–24 month	68				
> 24 month	48				
No. of previous infection control surgery	3.5 times	2-18	2.68	0.22	3.15–4.04
C-M classification	III: 28				
	IV: 112				
No. of microorganism	Culture negative : 20				
	Monomicrobial : 81				
	Polymirobial: 39				
Presence of P.aeruginosa	23				
Presence of anerobes	26				
Presence of Gram negative organism	42				
**Bone grafting environment**					
Type of defect	Segmental :110				
	Wedged : 30				
Length of defect	90.1 mm	30.3–219.0	41.2	3.48	83.13–96.63
< 50 mm	25				
50-100 mm	67				
> 100 mm	48				
Soft tissue coverage	Flap coverage: 70				
	None: 70				
Infected previous flap	23				
Single vessled limb	29				
No. of stages before Bone grafting	2.5	1-7	1	0.1	2.34–2.69
Duration of induced membrane	129.5 days	42–375	57.3	4.84	120.45–139.22
< 90 days	32				
90–120 days	38				
> 120 days	70				
Fixation construct	Nailing : 69				
	Plating : 34				
	Combined : 37				
**Details of bone grafting**					
Property of bone grafts	Pure autogenous bone graft:70				
	Mixed graft with DBM:70				
Percentage of DBM (%)	35.3	10.3–50.0	11.4	2.84	32.63–37.97
Addition of BMP-2	14 cases				
Circumferential Bone grafting	55 cases				
Mean operation time	215 min	62–445	71.5	6.04	203.48–225.72
**Surgical procedure**					
Deviation of protocol	41				
Surprise Positive Culture	29				
Abnormal ESR before BG	84				
Abnormal CRP before BG	16				

S.D.: Standard Deviation, S.E.: Standard Error, C.I.: Confidence Interval, BMI: Body Mass Index, ASA classification: American Society of Anesthesiologists physical status classification, DBM: Demineralized Bone Matrix.

**Table 2:** Isolated microorganisms.

Microorganism	
**Single Microorganism**	81 (57.9%)
MRSA	25
MSSA	13
MRCN	13
* Pseudomonas aeruginosa*	11
* Enterobacter cloacae*	4
* Enterococcus faecali*s	4
MSSE	2
* Staphylococcus lugdunensis*	2
* Serratia marcescnes*	1
* E. coli*	1
* Burkholderia arboris*	1
* Enterobacter hormaechei*	1
* Corynebacterium striatum*	1
* Staphylococcus caprae*	1
* Bacteriodes fragilis*	1
**Poly-Microorganism**	39 (27.9%)
2	25
3	12
> 4	2
**Culture negative infection**	20 (14.3%)

MRSA: methicillin-resistant staphylococcus aureus, MSSA: methicillin-susceptible staphylococcus aureus, MRCN: methicillin- resistant coagulase negative streptococcus epidermidis, MSSE: methicillin-susceptible streptococcus epidermidis.

### Primary outcome

**Figure 5:**
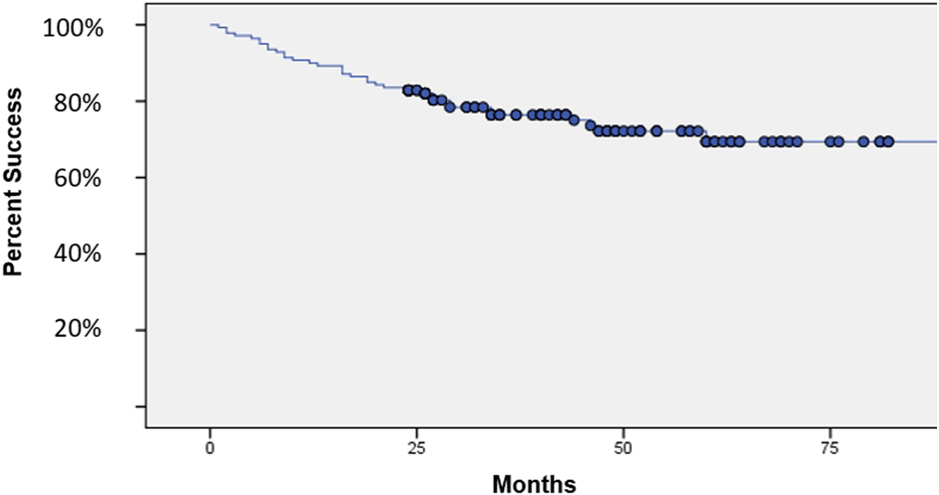
Kaplan–Meier curve showing success rate of the PDC based, multi-staged IMT with the end point for recurrence of infection. The success rate of multi-staged IMT at 24 months after bone grafting was approximately 80%.

A total of 140 patients had follow-up of at least 2 years after bone grafting. The primary success rate at 2 years was 75% (105/140). The mean infection-free interval was 45.3 months (range 24–84). The primary success rate of tibia and femur was 71.1% (69/97) and 83.7% (36/43), respectively. After excluding all “deviated protocol” cases, the success rate of our multi-staged IMT was 82.8% (82/99), (Tibia: 80.6% (54/67), Femur: 87.5% (28/32), respectively). The mean LEFS score in success group before and after the treatment were 12.1 ± 8.5 and 56.6 ± 9.9, respectively. Infection recurrence after final bone grafting occurred in 35 patients. The mean time of recurrence was 18.5 months (range 1–49 months) after final bone grafting. (Fig. 5).

### Secondary outcome—which factors determined infection recurrence?

Univariate cox regression analysis showed that the following variables were associated with ROI: “Type of soft tissue coverage,” “Number of stages before bone grafting,” “Infected previous free flap,” “Surprise positive culture,” “Deviation of protocol,” and “Elevated ESR before bone grafting”. Multivariate cox regression analysis identified the following variables as independent risk factors associated with ROI: “Infected previous free flap,” “Surprise positive culture,” “Deviation of protocol,” and “Elevated ESR before bone grafting” (Table 3).

**Table 3:** Risk factor analysis of Recurrence of Infection.

Variables	Univariate cox regression		Multivariate cox regression	
	HR (95% CI)	*p* value	HR (95% CI)	*p* value
Type of soft tissue	2.113 (0.986–4.531)	0.0445	1.537 (0.629–3.757)	0.345
Number of stages before BG	1.437 (1.116–1.85)	0.005	1.083 (0.845–1.387)	0.5284
Infected previous free flap	2.38 (1.19–4.761)	0.0142	3.14 (1.393–7.077)	0.0058*
Surprise culture positive	4.736 (2.359–9.507)	0.0001	3.608 (1.733–7.514)	0.0006*
Deviation of protocol	3.341 (1.703–6.553)	0.0004	2.585 (1.295–5.163)	0.0071*
Elevated ESR before BG	2.525 (1.142–5.581)	0.0221	2.24 (1.007–4.98)	0.0479*

*Significant at *p* value < 0.05.

BG: bone grafting.

## Discussion

**Figure 6:**
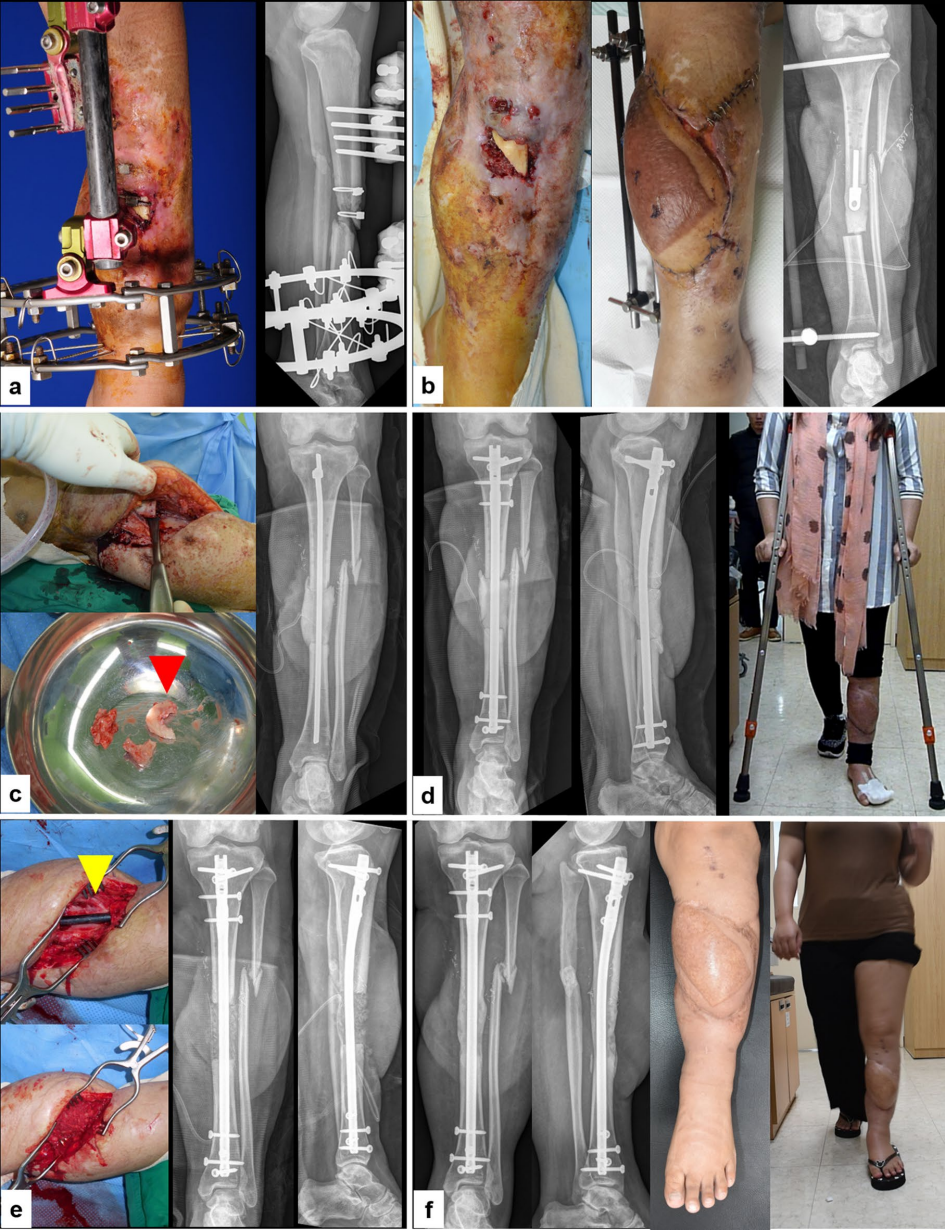
(**a**) A 24-year-old female patient sustained a recalcitrant infected nonunion of the left tibia. (**b**) According to the Multi-staged IMT protocol, we performed thorough debridement, placed an antibiotic-loaded PMMA spacer, and provided temporary bony stabilization. Soft tissue defects were covered using an anterolateral thigh flap (stage 1). (**c**) As PDCP had been identified at EMP and IMP (pseudomonas and MRSA), targeted debridement at proximal area of bone defect (red arrow head, sequestrum) and temporary fixation using antibiotics loaded PMMA coated intramedullary rod was performed (repetitive stage1). (**d**) After confirming all PDCN at previous stage, definitive fixation using IM nail was performed. An antibiotic-loaded PMMA spacer was reinserted into the bone defect for staged bone grafting to confirm that the infection had been eradicated (stage 2). Prompt partial weight bearing exercise was allowed after definitive fixation. (**e**) The 6-cm sized segmental bone defect was filled with autogenous bone (45 g) and DBM (15 g) in induced membrane chamber (yellow arrow head) at 16 weeks after stage 2 (stage 3). (**f**) Two years follow up radiographs show complete consolidation of the grafted bone without recurrent infection. The injured limb recovered to full function.

In our study, the success rate of limb reconstruction of CSBD in patients with IN and PTOM is slightly lower than in previous reports. Xie et al. reported an overall cure rate of 87.74% after 16 months of follow-up among 424 cases of osteomyelitis of the extremities treated with IMT^18^. One meta-analysis reported a primary success rate of 82% when the Masquelet technique was used in the treatment of 427 cases including the treatment of tumor reconstruction, infection (osteomyelitis and infected nonunions), aseptic nonunions and post-traumatic defects across 17 studies^19^. These slight differences in success rates can be attributed to several causes, including differences in the bone defects and anatomical sites of treatment, such as the upper extremity and in non-long bone infections; the severity of the infection; and follow-up duration. Our study evaluated cases of recalcitrant long bone infections of the lower extremity with a mean duration of 49 months before presentation to our institution, with a Cierny-Mader classification of III or IV that underwent at least two failed treatments at outside hospitals. All reasons why our infection eradication outcomes differed from those reported in previous case series using the IMT. However, our success rate of the 99 cases that did not deviate from our protocol, demonstrated similar rates (82.8%) from previous studies (Fig. 6).

Our results demonstrate how a PDC-based, multi-staged IMT is a promising treatment modality for recalcitrant IN or PTOM in the lower extremity by addressing residual infection, bony deformity, weight-bearing requirements, disuse osteopenia, soft tissue atrophy, and bone defects in a systemic stepwise fashion.

Our protocol emphasizes additional debridements and intraoperative cultures after these serial debridements. There is some debate as to how many additional debridements are needed to manage the infection. Although cases of false positive intraoperative cultures due to contamination have been reported^20^, the cultures and histological findings of the resected margins must be heavily considered when diagnosing residual osteomyelitis^21,22^. In our protocol, a PDCP indicated residual infection, thereby enabling targeted debridement for the next procedure. To reduce the likelihood of contamination, we changed all surgical drapes and instruments frequently.

Our treatment protocol is based on tissue cultures. Previous studies have reported low sensitivity of tissue culture results between 30 and 61%. To overcome the low detection rate of tissue culture, other methods such as PCR or inflammatory cell counts (PMN cell counts), next-generation sequencing are suggested in the literature. However, these methods do not have clear evidence yet or clinical application is limited^10,23^. In our protocol, to increase the detection rate of microorganisms, we tried to take tissue samples from multiple sites including PD cultures, implant sonication fluid cultures were taken, and these cultures were taken in each stage of the protocol.

Use of antibiotics plays a critical role in treating bone infections. As we mentioned, targeted antibiotics were administered for at least 6 weeks. Consultation with infectious disease specialists regarding the culture results, type of host, intraoperative findings is essential in selecting the appropriate antibiotics. Coadministration of rifampicin in the setting of retaining implant can be very effective against staphylococcal biofilm^24^. Also, longer duration upto 12 weeks with implant retention is suggested according to the recommendations from fracture-related infection expert group^25^.

Treatment of infected bone defects using induced membrane technique originally consisted of two stages as reported by Masquelet, et al. On the contrary, our protocol consists of three stages. By implementing definitive fixation (2^nd^ Stage) and bone grafting (3^rd^ Stage) as separate stages, we were able to confirm eradication of the infection at multiple time points prior to final bone grafting. Additionally, by creating a stable fixation construct at the 2^nd^ stage of the protocol, early range of motion and partial weight bearing was permitted following stage 2, as the PMMA could help to bear the load of the body weight. Another potential benefit to having separate stages for fixation and bone grafting is the ability to perform complex deformity corrections in a staged manner to decrease the surgical time required when combining fixation and bone grafting. In spite of these advantages of three staged protocol, we suggest that reducing the stages or combining 2nd and 3rd stage should be considered when the PD cultures are negative to reduce the burden on the patients.

Although previous reports have demonstrated that patients who needed soft tissue reconstruction with free flap coverage were more likely to have postoperative complications^18,26^, a thorough surgical debridement of infected tissue following a systematic soft tissue reconstruction is critical to achieve favorable results^2,27^. Type of soft tissue coverage was not statistically significant as a solitary risk factor in the multivariate regression model. However, among the 23 infected previous flaps, the ROI increased to 56.5% (13/23), and this variable was proven to be a solitary risk factor of ROI in our cohort (HR: 3.14, *p* = 0.0058). Recurrence of infection after an infected previous free flap can attributed to several factors. All these patients had initial free flaps at outside hospitals and were treated initially by limited debridement without a systematic protocol. These 23 patients with infected previous free flaps had a high ROI (13/23) possibly due to less aggressive excisional debridement for fear of compromising the flap and flap blood supply.

Although the bioactivity of the membrane has been found to peak 6–8 weeks after PMMA cement insertion^28–30^, eradication of infection must be verified before bone grafting. It was suggested that clinical examination, trends in acute inflammatory markers (including ESR and CRP), and intraoperatively obtained specimens pertaining to culture and pathology should be considered before bone grafting^31^. In addition, the clinical value of ESR was found to be a sensitive, specific, and independent marker for osteomyelitis recurrence^32,33^. We also could prove that the case when bone grafting was performed with elevated ESR prone to recur.

Detecting indolent infection is imperative during aseptic nonunion repair and revision arthroplasty^13,34^. Olszewski et al. identified 83 positive surprise cultures among 453 nonunion cases with suspected indolent infection-related, reporting an 80% survival rate following either residual infection or nonunion treated with an additional 6–8 weeks of antibiotics^13^. In the study by Tsukayama et al., the proportion of positive intraoperative cultures among 275 cases of revision hip arthroplasty for suspected aseptic loosening was 11%, with a 90% success rate after 6 weeks of antibiotics^35^. In our cohort of 140 cases, we found that 29 cases (20.7%) of intraoperative “surprise positive culture” progressed to the next treatment stage. Although we treated indolent infection with 6 weeks of antibiotics, only 14 cases (48.3%) were successfully treated. Hence, a “surprise positive culture” during our staged IMT protocol was associated with a 3.6-fold increased risk of recurrence (*p* = 0.0006). Contrary to prior reports on indolent infections, additional debridement must be considered when such positive cultures are found during staged reconstruction, as antibiotic suppression therapy may be insufficient.

“Deviation of protocol” was defined as an alteration of the treatment protocol with combining protocol stages, retaining implants after PDCP, and not returning to a previous stage of the protocol after PDCP. A total of 41 cases were identified as Deviation of protocol in our cohort. These deviations in protocol were independent risk factors for ROI. Of these 41 cases, 18 resulted in ROI (43.9%). A deviation from our protocol resulted in a 2.6-fold higher risk of ROI (*p* = 0.0071). This demonstrates adhering to a systemized reconstruction protocol is crucial when treating recalcitrant chronic bone infections (Table 3).

The inherent limitations of any retrospective cohort study is also applied to the present study despite the data being collected prospectively. First, we did not consider cases in which patients underwent amputation preceding bone grafting for uncontrolled and disseminated infection. Second, while we identified four variables predicting ROI, it is likely that more exist. Third, it was difficult to conclude whether recurrence of infection was due to latent or new infection. Despite these limitations, this study includes a large cohort of patients with CSBD secondary to IN and PTOM, treated with a systematic, detailed protocol with clearly defined outcomes while elucidating factors which increase risk of infection recurrence and graft failure.

## Conclusion

The PDC based, multi-staged IMT protocol yielded promising success rates in limb reconstruction for the treatment of recalcitrant IN and PTOM. The recurrence of infection was influenced by Infected previous free flap, Surprise positive culture, Deviation of protocol, and Elevated ESR before bone grafting.

## Data Availability

All data generated or analysed during this study are included in this published article and uploaded as Supplementary material.
